# A Virtual Versus an Augmented Reality Cooking Task Based-Tools: A Behavioral and Physiological Study on the Assessment of Executive Functions

**DOI:** 10.3389/fpsyg.2019.02529

**Published:** 2019-11-14

**Authors:** Irene Alice Chicchi Giglioli, Cristina Bermejo Vidal, Mariano Alcañiz Raya

**Affiliations:** Instituto de Investigación e Innovación en Bioingeniería (I3B), Universitat Politècnica de València, Valencia, Spain

**Keywords:** executive functions, ecological validity, virtual reality, augmented reality, behavioral performance, physiological signals

## Abstract

Virtual reality (VR) and augmented reality (AR) are two novel graphics immersive techniques (GIT) that, in the last decade, have been attracting the attention of many researchers, especially in psychological research. VR can provide 3D real-life synthetic environments in which controllers allow human interaction. AR overlays synthetic elements to the real world and the human gaze to target allow hand gesture to act with synthetic elements. Both techniques are providing more ecologically environments than traditional methods, and most of the previous researches, on one side, have more focused on the use of VR for treatment and assessment showing positive effectiveness results. On the other, AR has been proving for the treatment of specific disorders but there are no studies that investigated the feasibility and effectiveness of AR in the neuropsychological assessment. Starting from these premises, the present study aimed to compare the performance and sense of presence using both techniques during an ecological task, such as cooking. The study included 50 cognitively healthy subjects. The cooking task consisted of four levels that increased in difficulty. As the level increased, additional activities appeared. The order of presentation of each exposure condition (AR and VR) was counterbalanced for each participant. The VR-cooking task has been performed through “HTC/VIVE” and AR through “Microsoft HoloLens.” Furthermore, the study recorded and compared the psychophysiological changes [heart rate and skin conductance response (SCR)] during the cooking task in both conditions. To measure the sense of presence occurring during the two exposure conditions, subjects completed the Slater-Usoh-Steed Questionnaire (SUSQ) and the ITC-Sense of Presence Inventory (ITC-SOPI) immediately after each condition. The behavioral results showed that times are always lower in VR than in AR, increasing constantly in accordance with the difficulty of the tasks. Regarding physiological responses, the findings showed that AR condition produced more individual excitement and activation than VR. Finally, VR was able to produce higher levels of sense of presence than AR condition. The overall results support that VR currently represents the GIT with greater usability and feasibility compared to AR, probably due to the differences in the human–computer interaction between the two techniques.

## Introduction

Virtual reality (VR) and augmented reality (AR) are two novel graphics immersive techniques (GIT) that, in the last decade, have been attracting the attention of many researchers, especially in the fields of psychology and education (Chicchi Giglioli et al., 2015; Negut et al., 2016; Fleming et al., 2017; Cipresso et al., 2018; Jensen and Konradsen, 2018; Ventura et al., 2018; Germine et al., 2019). On one side, VR is an interactive and advanced computer technology that it can create real-simulated three-dimensional (3D) environment. Technologically, VR provides a wide field of view (FOV – the area angular size allowed to a user to see a scene) around 100° and the human–computer interaction can be ensured by various devices, such as head-mounted display (HMD) for the visual stimuli, headphone for the acoustic stimuli, controllers for hand interaction. These allow users to navigate and interact with the virtual environment, being felt them totally immersed in the virtual world. The accurate real-simulated 3D environment and the technological presence can help users to generate a sense of presence, defined as the feeling to “being in” the virtual environment (Gregg and Tarrier, 2007; Slater, 2009; Parsons, 2015; Valmaggia et al., 2016; Freeman et al., 2017). On the other, AR is a recent technology in which synthetic elements are incorporated in the physical world adding information to the users (Chicchi Giglioli et al., 2015; Ventura et al., 2018). The FOV is narrower than VR, included between 35° and 45° and the interaction is ensured by various sensors integrated into the headband, like cameras that, through the human gaze to target, allow the real hands’ interaction with the synthetic elements. AR, like VR, aims to provide high visual realism, fidelity of the experience, and presence, highly similar to the real one and adding real objects/information to real world. Visual realism and fidelity can depend on the FOV, accuracy, complexity of the systems, as well as on the user’s interaction fidelity. Regarding the visual realism and fidelity, a wider FOV allows the user to see more of the scene at once and to use peripheral vision, while a narrower FOV, as in AR systems, may reduce distraction in the periphery and allow the user to focus on the area of interest in the scene (Ragan et al., 2010, 2012; McMahan et al., 2012). Furthermore, high accuracy and complexity on graphics can enhance the level of fidelity of the experience, allowing transferring the VR/AR learned behaviors in real-world or allowing to perform in the AR/VR world, as if the user were in the real-life (Dunkin et al., 2007; Seymour, 2008; Saposnik et al., 2010). Finally, interaction fidelity supposed that more is natural the interaction, higher is the fidelity (McMahan et al., 2012). However, comparison studies on different hand controllers showed that the more familiar, and less natural type of controller provided a best performance, although the participants appreciated the more natural interaction (McMahan et al., 2010). All these features are able to generate immersed and the psychological state to be present in the virtual and augmented environments (Slater, 2009). A valid and reliable measure for the sense of presence is the ITC-Sense of Presence Inventory (ITC-SOPI; Lessiter et al., 2001) that assess four dimensions: sense of physical space, engagement (E), ecological validity (EV), and negative effects (NE). Tang et al. (2004), compared the sense of presence between a VR and an AR environment, showing significantly higher score for sense of physical space for AR, and no significant differences in the other three dimensions, although all means were higher in the AR than VR condition.

According to this, at present, both techniques are providing advantages along with traditional scientific research procedures, providing accurate real-simulated stimuli control and behavior measurement of reactions times and scores and allowing researcher to address issues that would simply be difficult to pose in natural environments (Bohil et al., 2011; Germine et al., 2012; De Leeuw, 2015; Reimers and Stewart, 2015). In psychology both technologies have been extensively explored in the treatment of certain disorders, such as phobias, allowing patients learning and repeating new behaviors to cope with fearful stimuli in safe and reactive environments generating effectiveness in behavioral changes in real contexts (Chicchi Giglioli et al., 2015; Suso-Ribera et al., 2018; Ventura et al., 2018). In psychological assessment, conversely, several VR applications have been developed for neuropsychological evaluation in order to improve the EV of them (Pugnetti et al., 1998; Ku et al., 2003, Ku et al., 2004; Rizzo et al., 2004; Rand et al., 2007, 2009; Díaz-Orueta et al., 2012; Henry et al., 2012; Parsons et al., 2013; Cipresso et al., 2014; Díaz-Orueta et al., 2014). Traditional neuropsychological assessment consists of performance-based approach, involving paper-and-pencil and/or computerized tests, to assess a variety of cognitive processes, such as attention, memory, inhibition control, planning, cognitive flexibility, and the higher-order system of executive functions, that govern the cognitive processes to goal-directed and adaptive behaviors. These tests consist of a set of predefined and abstracts’ stimuli delivered in a controlled setting that have proved moderate level of EV in predicting real-functional performance (Elkind et al., 2001; Chaytor and Schmitter-Edgecombe, 2003; Chaytor et al., 2006). For example, the Tower of London is a neuropsychological measure for the assessment of executive functioning, specifically related to planning abilities, in which a target configuration of colored beads are presented to the participant and he/she is asked to compute the minimal number of steps (ranging from 1 to 5) to reach a target configuration. This test is a reliable and valid measure but it is abstract and decontextualized from the real-life activities.

In order to improve similarity between tests and real-life activities, several VR environments have been developed such as virtual mall/supermarket (Rand et al., 2007, 2009; Cipresso et al., 2014), and classroom (Rizzo et al., 2000, Rizzo et al., 2009; Díaz-Orueta et al., 2014). For example, Cipresso et al. (2014) tested a virtual supermarket in which participants (patients with normal cognition, patients with mild cognitive impairments and cognitively healthy subjects) had to complete four shopping tasks. Findings revealed that the virtual shopping task was able to discriminate the performance among the three groups and that the virtual supermarket was more sensitive than traditional assessment in detecting cognitive impairments. Furthermore, a recent meta-analytic review (Negut et al., 2016) on VR applications in neuropsychological assessment showed moderate sensitivity and effect size in detecting cognitive impairments by comparing performance between health subjects and patients using both VR applications and traditional measures.

Despite the opportunities, that VR has been providing in psychological assessment, to our knowledge no previous studies have investigated the differences in behavioral responses to ecological tasks presented through AR compared to other methods – particularly VR.

Finally, both systems are also compatible with other neuroscientific tools such as wrist devices able to measure changes in electrodermal activity (EDA) and heart rate variability (HRV) (Poh et al., 2010; Garbarino et al., 2014). EDA and HRV showed consistent results with cognitive and information processing (Dawson et al., 2007; Sequeira et al., 2009) and can provide, together with behavioral data, implicit and objective responses to changing during activities.

Starting from these premises, the first aim of this study was to analyze and compare behavioral and physiological data collected before, during and after performing a cooking task in VR and AR environments. Second, the study aimed to determine the degree of presence, or the feeling of “being there,” that produced VR through the “HTC Vive” and AR through “Microsoft HoloLens.”

## Materials and Methods

### Participants

The experimental sample included 50 healthy individuals (16 males and 34 women). Participants were recruited through local advertisement among college students and workers of the Polytechnic University of Valencia. The mean age was 25.96 ± 6.51. To be included in the study, participants were required to have a score higher than 24 in the “Mini-Mental State Examination” (MMSE) (Folstein et al., 1975). Before participating in the study, each participant was provided with written information about the study and required to give written consent for inclusion in the study. The study received ethical approval by the Ethical Committee of the Polytechnic University of Valencia. Table 1 includes the main sociodemographic data, such as age, gender, and education.

**TABLE 1:** Sociodemographic data of the participants (*n* = 50).

**Sociodemographic data**	**Mean (SD)**	**[Range]**
Age	25.96 (6.51)	[18–48]
Gender (man/woman)	16/34	
Education (high school/bachelor degree/postgraduate degree)	6/28/16	

### Psychological Assessment

Before the experimental session, the following questionnaires were administered to each participant:

• Attentional Control Scale (ACS) (Derryberry and Reed, 2002) is used to evaluate the attentional control and higher scores show a great ability to maintain voluntarily attention in a task, while low values are related to greater attention stiffness.
• Barratt Impulsiveness Scale (BIS-11) (Barratt, 1959; Oquendo et al., 2001) is a measure of impulsiveness and a score of 72 or more means that the individual is highly impulsive. Between 52 and 71 should be considered within the normal limits of impulsivity. Below 52 represents a subject excessively controlled.
• Cognitive Flexibility Scale (CFS) (Martin and Rubin, 1995) consists of 12 questions that are scored on 6 points Likert-scale; a score of 60 or more indicates that the individual has a high cognitive flexibility.

Furthermore, participants completed a total of 5 standard tasks (ST): Dot Probe Task (DOT) version published by Miller and Fillmore (2010); Go/NoGo Task (Fillmore et al., 2006); Stroop Test (Stroop, 1992); Trail Making Task (TMTA-B), paper-and-pencil-based version published by Reitan (1958); and Tower of London – Drexler (TOLDX; Culberston and Zillmer, 1999). The ST were randomly presented and performed on a personal computer. Neuropsychological data performance of the participants are reported in Table 2.

**TABLE 2:** Mean (*M*) standard deviation (*SD*), and range (Min., Max.) of values for questionnaires and standardized tasks.

**Variables**	***M***	***SD***	**Min.**	**Max.**
CFS	47.36	6.59	34	64
BIS_Cognitive	18.94	2.68	14	25
BIS_Motor	22.14	4.99	14	38
BIS_No Planning	24.16	4.87	15	39
BIS	65.24	9.46	50	91
ACS	55.44	8.07	41	69
DOT_TT	159.01	5.31	151.28	175.00
DOT_CA	0.99	0.01	0.96	1.00
DOT_LT	0.46	0.06	0.36	0.61
GONOGO_CA	0.99	0.02	0.93	1.00
GONOGO_LT	0.41	0.04	0.31	0.53
TMT_TTA	35.54	7.18	22.81	56.08
TMT_TTB	54.32	16.57	28.92	134.08
TMT_CAA	25.00	0.00	25.00	25.00
TMT_CAB	25.00	0.00	25.00	25.00
TOL_TT	436.64	604.16	124.58	4492.58
TOL_CA	9.56	0.99	5.00	10.00
TOL_TS	25.44	3.46	14.00	29.00
TOL_ET	20.24	7.37	7.18	42.44
STROOP_TT	3208.61	19516.38	75.09	137260.17
STROOP_CA	0.99	0.03	0.82	1.00
STROOP_LT	1.27	0.22		

*TT = total time; CA = correct answers; LT = latency time; TS = total score; ET = execution time.*

After each exposure condition, the following presence questionnaires were administered to each participant:

• Slater-Usoh-Steed Questionnaire (SUSQ) (Slater and Steed, 2000): This *post hoc* test consists of three questions that are evaluated on a scale of 7 points. The items evaluate the sensation of being in the environment, the extent to which the medium becomes the dominant reality and the magnitude in which it is remembered as a “place.”
• ITC-SOPI (Lessiter et al., 2001): This test consists of 42 items, evaluated on a scale of 5 points, and evaluates four dimensions of presence: the sense of physical space or spatial presence (SP), E, EV, and NE.

Descriptive data on presence are reported in Table 3.

**TABLE 3:** Mean (*M*) and standard deviation (*SD*) of values for presence questionnaires.

**Variables**	***M***	***SD***	**Min.**	**Max.**
SUSQ_AR	4.11	1.65	1.33	7
SUSQ_VR	5.85	1	3	7
SOPI_SP_AR	3.29	0.64	1.83	4.61
SOPI_E_AR	3.6	0.69	2.08	4.69
SOPI_EV_AR	3.21	0.86	1.6	5
SOPI_NE_AR	1.7	0.64	1	3.4
SOPI_SP_VR	3.81	0.57	2.39	4.67
SOPI_E_VR	4.21	0.49	3.08	5
SOPI_EV_VR	3.93	0.73	1.8	5
SOPI_NE_VR	1.52	0.51	1	3.25

### Physiological Assessment

At the beginning and during the experimental session, skin conductance response (SCR) and HRV were recorded to obtain subjects’ physiological responses to VR and AR cooking task. SCR and HRV are considered indexes of arousal responses (Boucsein, 1992). The physiological signals were acquired using Empatica E4 device, including E4 Manager software to record and export raw signals. The sampling frequency in the SCR signal was acquired at 4 Hz, and 64 Hz for HRV, inside a window time from 1 to 2.5 s with an amplitude >0.01 μS (microvolts).

### The Cooking Task

The virtual and augmented system was developed using Unity 5.5.1f1 software, applying c# programing language using the Visual Studio tool. Participants performed the virtual cooking task wearing an HMD device (HTC VIVE^1^) and through two hand controllers, and the augmented cooking task using Microsoft HoloLens^2^. The AR experience was performed in a real kitchen in which the augmented synthetic objects appeared in front of the subject according to the subjective human gaze. The interaction in AR was ensured by various sensors integrated into the headband, like cameras that, through the human gaze to target, allow the real hands’ interaction with the synthetic elements.

Before the VR and AR virtual cooking task, participants performed two introductory tasks (tutorial), one for each technology, in order to learn the main body movements and hands’ interactions useful to perform the virtual cooking task. The tutorial consisted of a simulated task, similar to the virtual cooking task. In both conditions, body movements were real in the physical space and hands’ interaction in the VR was performed through the use of two controllers and in AR, participant interacted with objects with their own hands. Participants could train for as long as necessary, according to the needs of each one. When they felt confident about body and hand movements and interactions, a button pulsed to start the virtual cooking task.

**FIGURE 1:**
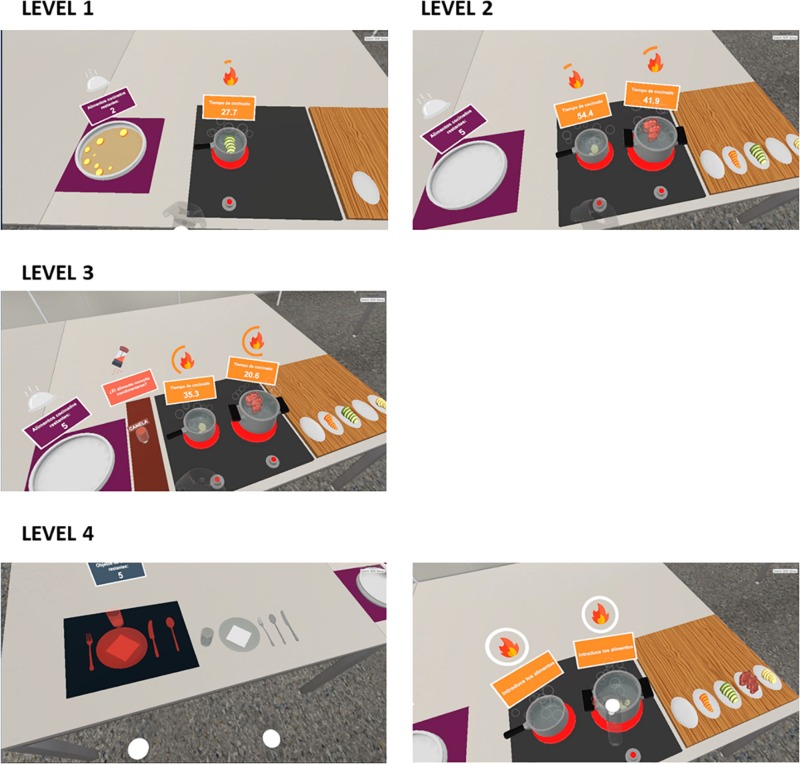
The cooking task levels.

The virtual cooking task consisted of four levels of difficulty, involving the abilities to pay attention, planning, and shifting. All were based on cooking a series of food in a set time, avoiding burning (in which the ingredient was in the fire more than the set time) or cooling them (switch off the glass-ceramic switch or remove the food from the pan during cooking). As the level increased, additional activities appeared (Figure 1). In the first level, participants had to cook three foods in one cooker on 2 min; in the second level, they had to cook 5 foods on 2 cookers in 3 min; in the third level, a dual-task should be performed: (a) 5 foods should be cooked on 2 cookers in 4 min; (b) during the cooking, participants should add the right dressing to the foods; in the last level, another dual-task has been proposed: (a) participants should cook 5 foods in 2 cookers in 5 min; and (b) they should set a table. Each food should be cooked in a scheduled time, as well as the level had a limit time that appeared all the time in the virtual and augmented environment. When the food was cooked, it had to be removed from the pan, turning off the cooker and placed in the dish. The main aim of each level was to cook the foods in the scheduled time without burning and letting them cool. Burning a food means by not taking it out of the pan, or turning the burner off, after the predefined cooking time. Cooling a food means left the food in the pan to cool down after it was cooked. The virtual system gathered various time/performance data for each subtask, including total times, burning times, and cooling times.

**FIGURE 2:**
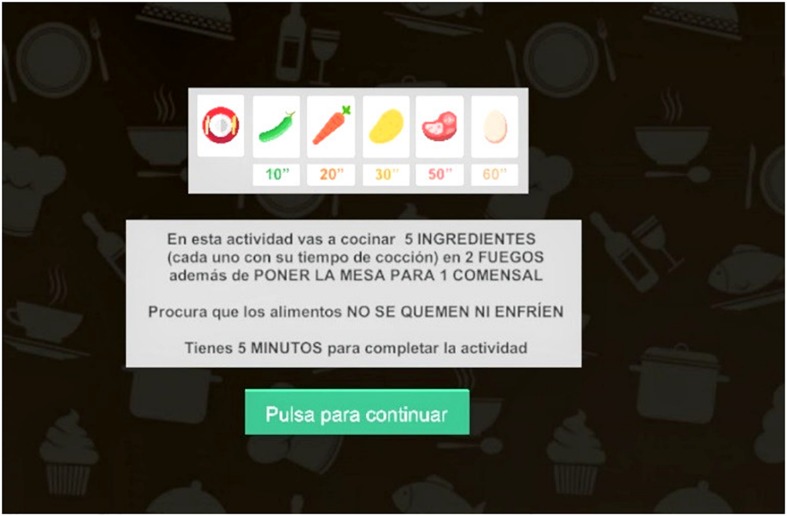
The cooking task instructions.

Participants exceeded the following level when they have cooked all the foods, completing the level. Before each level, instructions, explaining what activities participants had to be carried out, what time they had to do it, times for each food and remembering to cook foods without burning and letting them cool, have been showed (Figure 2).

### Experimental Procedure

The order of presentation of each exposure condition (AR and VR) was counterbalanced for each participant. Before the beginning of the experiment, participants were administered the MMSE and standard questionnaires (ACS, BIS, CFS) and tasks (DOT, GoNoGo, Stroop, TMTA-B, TOL). Once this first phase was completed, we recorded 3-min of EDA and HRV baseline, asking to participants to stay completely relaxed during the recording. Once the physiological baseline was recorded, the experimental session started, and EDA and HRV were continuously recorded until the end of the experiment. To measure the sense of presence occurring during the two exposure conditions, subjects completed the SUSQ and the ITC-SOPI immediately after each condition.

### Statistical Analyses

The analyses were performed using SPSS version 22.0 (Statistical Package for the Social Sciences for Windows, Chicago, IL, United States) for PC. The biosignals’ processing and computation were analyzed using MATLAB and Ledalab programs. First, we verified the assumptions of normality applying Kolmogorov–Smirnov and the internal consistency of the scales was assessed via Cronbach’s alpha.

Second, it has been verified the normal cognitive functioning and the physiological health (SDNN and rMSSD of HRV values) of the subjects.

Next, four paired *t*-tests were conducted to compare behavioral, physiological data (SCR and HR), and sense of presence responses in AR and VR conditions. The level of significance was set at α = 0.05.

## Results

The assumption of normality was confirmed (Kolmogorov–Smirnov *p* > 0.05) and the internal consistency of the self-report scales has been measured (Cronbach’s alpha αattention = 0.819, αcognitive flexibility = 0.765, αimpulsiveness = 0.785; αSUSQ_AR = 0.907; αSUSQ_VR = 0.702; αITC-SOPI_AR = 0.946; αITC-SOPI_VR = 0.937; bootstrap 95%).

Regarding the cognitive functioning (Table 2), the mean total score on cognitive flexibility showed that the subjects had a high cognitive flexibility (CFS TOTAL = 47.36; normal range: 10–60); the mean total value on impulsivity (BIS TOTAL = 65.21) is within the normal limits of impulsivity (normal range: 52–71); and for attentional control, a very high mean score was obtained (ACS TOTAL = 55.44), indicating that subjects were able to voluntarily control their attention. Table 2 also reports the descriptive data on standardized tasks.

Focusing on health at physiological level, the values of beats per minute (BPM) at baseline and during the tasks are in the normal range of 60–100 beats/min. Also, SDNN values indicate that participants are not in danger of suffering from any cardiac episode since the data is greater than 100 ms, while the rMSSD are also in the normal range (greater than 25 ms) (Macías, 2016) (Table 4).

### Behavioral Responses to Cooking Task

Regarding performance, Table 5 shows the mean and standard deviations of behavioral values of the cooking task for both conditions.

**TABLE 4:** Mean and standard deviation of heart rate (HR) SDNN and rMSDD values for condition.

	**AR**		**VR**	
		
	***M***	***SD***	***M***	***SD***
HR_SDNN_Baseline (ms)	118.09	50.48	126.16	44.18
HR_SDNN_Postline (ms)	110.11	48.11	123.44	41.89
HR_rMSSD_Baseline (ms)	146.71	75.69	164.81	60.75
HR_rMSSD_Postline (ms)	137.90	76.84	162.02	64.57
HR_SDNN_Level 1 (ms)	180.06	76.17	216.94	57.48
HR_rMSSD_Level 1 (ms)	239.58	110.38	295.25	84.80
HR_SDNN_Level 2 (ms)	185.30	85.79	233.99	62.46
HR_rMSSD_Level 2 (ms)	243.30	114.33	317.66	94.45
HR_SDNN_Level 3 (ms)	179.48	81.48	234.20	53.50
HR_rMSSD_Level 3 (ms)	242.90	107.34	315.49	75.58
HR_SDNN_Level 4 (ms)	113.27	45.87	253.63	52.69
HR_rMSSD_Level 4 (ms)	141.99	74.77	341.28	76.68
HR_SDNN_Level 4 (ms)	113.27	45.87	253.63	52.69
HR_rMSSD_Level 4 (ms)	141.99	74.77	341.28	76.68

**FIGURE 3:**
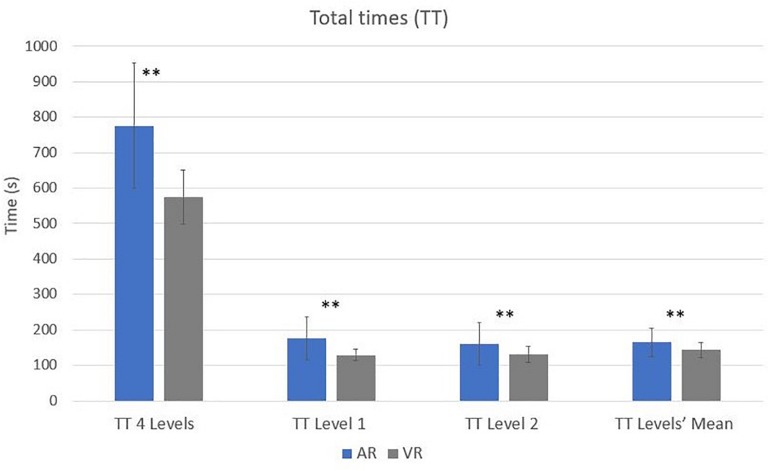
Paired *t*-test significant differences between conditions for total times (^∗^*p* ≤ 0.05, ^∗∗^*p* ≤ 0.01).

**FIGURE 4:**
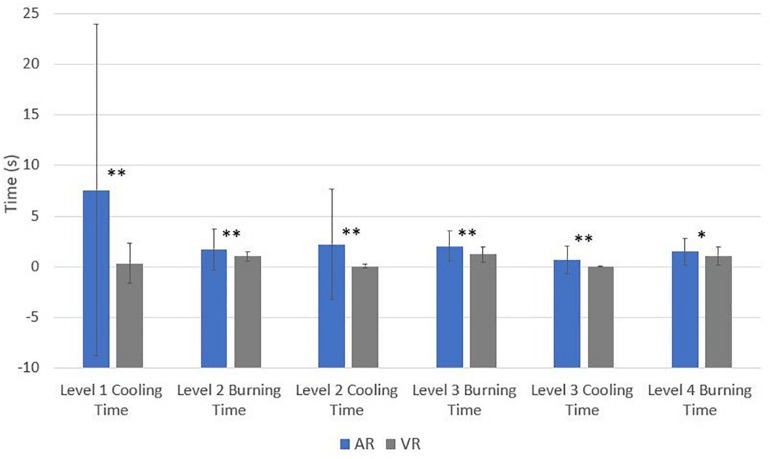
Paired *t*-test significant differences between conditions for cooling and burning time (^∗^*p* ≤ 0.05, ^∗∗^*p* ≤ 0.01).

A paired *t*-test was conducted to compare behavioral responses in AR and VR conditions. There were significant differences in the scores for the total four levels’ time between AR (*M* = 776.07, SD = 176.89) and VR (*M* = 574.13, SD = 76.22) conditions; *t*(49) = 7.75, *p* = 0.00, as well in the total time of level 1 in AR (*M* = 177.58, SD = 60.47) and VR (*M* = 129.78, SD = 16.45) conditions; *t*(49) = 3.08, *p* = 0.00 and in the level 2 in AR (*M* = 160.60, SD = 60.33) and VR (*M* = 132.09, SD = 22.30) conditions; *t*(49) = −3.08, *p* = 0.00 (Figure 3). Regarding cooling times significant differences between conditions have been found at level 1 [AR (*M* = 7.57, SD = 16.36), VR (*M* = 0.33, SD = 1.97); *t*(49) = 3.08 *p* = 0.00], level 2 [AR (*M* = 2.23, SD = 5.46), VR (*M* = 0.06, SD = 0.21); *t*(49) = −2.81, *p* = 0.01] and level 3 [AR (*M* = 0.66, SD = 1.37), VR (*M* = 0.01, SD = 0.05); *t*(49) = 3.37, *p* = 0.00]. Finally, significant differences on burning times have been found between conditions at level 2 [AR (*M* = 1.74, SD = 2.02), VR (*M* = 1.02, SD = 0.47); *t*(49) = 2.68, *p* = 0.01], level 3 [AR (*M* = 2.00, SD = 1.50), VR (*M* = 1.22, SD = 0.74); *t*(49) = −3.55, *p* = 0.00], and level 4 [AR (*M* = 1.48, SD = 1.36), VR (*M* = 1.06, SD = 0.91); *t*(49) = 2.12, *p* = 0.04] (Figure 4).

### Physiological Responses to the Cooking Task

#### Electrodermal Activity

**FIGURE 5:**
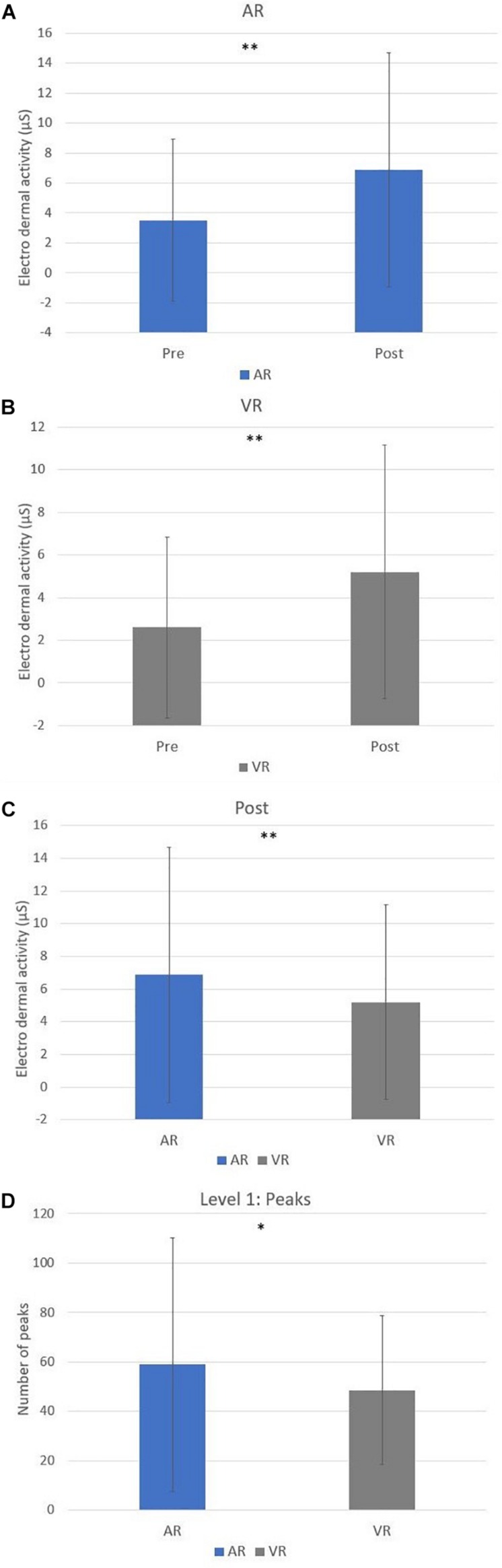
Paired *t*-test significant differences for EDA between AR and VR conditions: **(A)** EDA pre-post differences in AR; **(B)** EDA pre-post differences in VR; **(C)** EDA post- differences between AR and VR; **(D)** Activaction differences between AR and VR (^∗^*p* ≤ 0.05, ^∗∗^*p* ≤ 0.01).

First, Table 6 shows the mean and standard deviation of EDA values of the cooking task for both conditions. Second, a paired *t*-test was computed to compare physiological responses in AR and VR conditions. There was a significant difference in the scores for EDA for AR-pre (*M* = 3.51, SD = 5.42) and -post-task (*M* = 6.87, SD = 7.81) conditions; *t*(49) = −5.16, *p* = 0.00, as well for VR-pre (*M* = 2.60, SD = 4.25) and -post-tasks (*M* = 5.20, SD = 5.95) conditions; *t*(49) = −4.22. Another significant difference in the scores for EDA for AR-post (*M* = 6.87, SD = 7.81) and VR-post-task (*M* = 5.20, SD = 5.95) conditions; *t*(49) = −2.95, *p* = 0.00 has been found. Finally, there was a significant difference in the scores for number of peaks in the first level task between AR (*M* = 59.08, SD = 51.30) and VR (*M* = 48.52, SD = 30.07) conditions; *t*(49) = 2.01, *p* = 0.05 (Figures 5A–D).

**TABLE 5:** Mean and standard deviation of values for behavioral responses in AR and VR conditions.

	**AR**		**VR**	
		
	***M***	***SD***	***M***	***SD***
Total time four levels (s)	776.07	176.89	574.13	76.22
Total time Level 1 (s)	177.58	60.47	129.78	16.45
Level 1 burning time (s)	1.28	0.98	1.13	0.81
Level 1 cooling time (s)	7.57	16.36	0.33	1.97
Total time Level 2 (s)	160.60	60.33	132.09	22.30
Level 2 burning time (s)	1.74	2.02	1.02	0.47
Level 2 cooling time (s)	2.23	5.46	0.06	0.21
Total time level 3 (s)	165.47	50.62	159.22	31.75
Level 3 burning time (s)	2.00	1.50	1.22	0.74
Level 3 cooling time (s)	0.66	1.37	0.01	0.05
Total time level 4 (s)	158.26	48.85	154.68	55.62
Level 4 burning time (s)	1.48	1.36	1.06	0.91
Level 4 cooling time (s)	1.60	7.14	0.13	0.70
Total time levels’ mean(s)	165.48	39.98	143.94	21.92

**TABLE 6:** Mean and standard deviation of EDA values in AR and VR conditions.

	**AR**		**VR**	
		
	***M***	***SD***	***M***	***SD***
EDA_Baseline (μS)	3.51	5.42	2.60	4.25
EDA_Postline (μS)	6.87	7.81	5.20	5.95
EDA_TOT (μS)	1.94	3.17	2.12	3.58
EDA_SCR_TOT (μS)	0.11	0.10	0.12	0.15
EDA_SCL_TOT (μS)	1.85	3.09	2.00	3.46
EDA_N_PEAK_TOT	221.02	178.73	208.96	133.77
Task1_EDA (μS)	1.39	2.34	1.47	2.54
Level 1_SCR (μS)	0.09	0.09	0.10	0.12
Level 1_SCL (μS)	1.30	2.28	1.37	2.46
N_PEAK_Level 1	59.08	51.30	48.52	30.07
Level 2_EDA (μS)	1.70	2.71	1.73	3.03
Level 2_SCR (μS)	0.09	0.09	0.12	0.16
Level 2_SCL (μS)	1.61	2.65	1.61	2.90
N_PEAK_Level 2	51.82	43.54	50.10	31.99
Level 3_EDA (μS)	2.07	3.53	2.08	3.51
Level 3_SCR (μS)	0.09	0.11	0.13	0.18
Level 3_SCL (μS)	1.97	3.46	1.95	3.36
N_PEAK_Level 3	55.46	45.27	57.90	42.43
Level 4_EDA (μS)	2.52	3.89	3.00	6.55
Level 4_SCR (μS)	0.11	0.10	0.16	0.20
Level 4_SCL (μS)	2.42	3.81	2.83	6.40
N_PEAK_Level 4	54.66	46.13	52.44	33.26

No other significant differences in physiological activation during the four levels of the cooking task have been found.

#### Heart Rate Variability

Table 7 shows the mean and standard deviation of HRV values of the cooking task for both conditions (AR vs. VR).

**TABLE 7:** Mean and standard deviation for HRV values for AR and VR conditions.

	**AR**		**VR**	
		
	***M***	***SD***	***M***	***SD***
HR_Baseline (bpm)	79.57	13.43	81.93	8.09
HR_Postline (bpm)	81.20	14.07	81.41	6.22
HR_BeatPerMinute_Levels(bpm)	82.26	14.21	81.97	6.15
HR_BeatPerMinute_Level1 (bpm)	81.57	13.77	82.90	6.94
HR_BeatPerMinute_Level2 (bpm)	81.65	13.81	82.92	6.78
HR_BeatPerMinute_Level3 (bpm)	81.83	13.87	82.89	6.66
HR_BeatPerMinute_Level4 (bpm)	82.70	7.88	82.75	6.67

**FIGURE 6:**
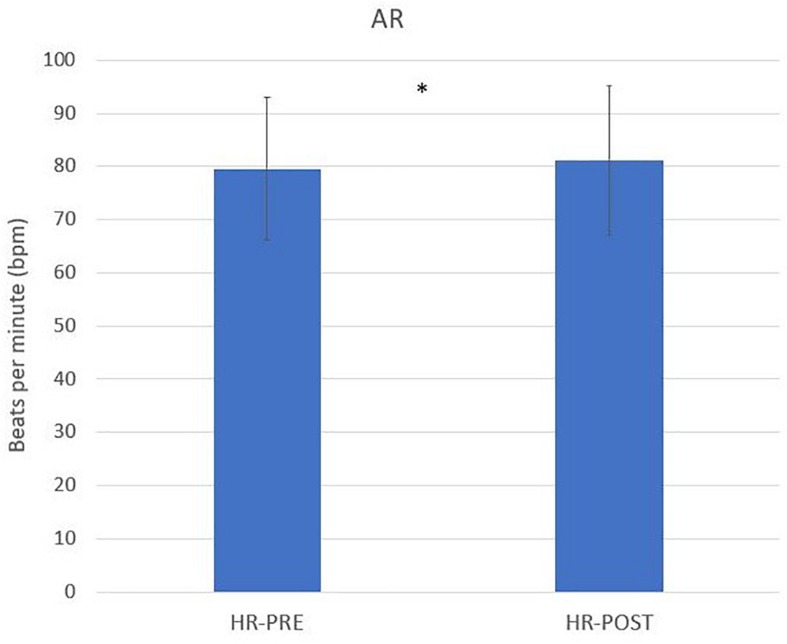
Paired *t*-test significant difference for HRV for AR-pre and -post-task (^∗^*p* ≤ 0.05, ^∗∗^*p* ≤ 0.01).

A paired *t*-test was computed to compare HRV in AR and VR conditions. There was a significant difference in the scores for HRV for AR-pre (*M* = 79.57, SD = 13.43) and -post (*M* = 81.20, SD = 14.07) task; *t*(49) = −1.97, *p* = 0.05 (Figure 6). No other significant differences in HRV during the four levels of the cooking task have been found.

### Sense of Presence

**FIGURE 7:**
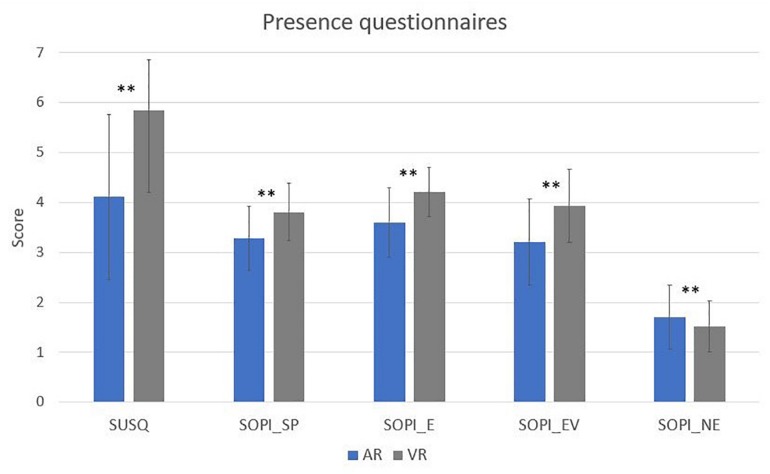
Paired *t*-test significant difference for presence questionnaires for AR and VR conditions (^∗^*p* ≤ 0.05, ^∗∗^*p* ≤ 0.01).

A paired *t*-test was computed to compare SUSQ and ITC-SOPI questionnaires in AR and VR conditions. Regarding the SUSQ, there was a significant difference in the scores for AR (*M* = 4.11, SD = 1.65) and VR (*M* = 5.85, SD = 1.00) conditions; *p* = 0.00. The ITC-SOPI showed significant differences between AR and VR in the four dimension of presence: SP [AR (*M* = 3.29, SD = 0.64); VR (*M* = 3.81, SD = 0.57) *p* = 0.00]; E [AR (*M* = 3.6, SD = 0.69); VR (*M* = 4.21, SD = 0.49) *p* = 0.00]; EV [AR (*M* = 3.21, SD = 0.86); VR (*M* = 3.93, SD = 0.73)]; and NE [AR (*M* = 1.7, SD = 0.64); VR (*M* = 1.52, SD = 0.51)] (Figure 7).

## Discussion and Conclusion

The first aim of this study was to analyze and compare behavioral and physiological data collected before, during and after performing the cooking task in VR and AR environments. The results on the behavioral data comparison showed that times are always lower in VR than AR, both for the times’ means of the four levels and in the specific levels. This may be because the interaction with VR is usually simpler; however, the VR and AR descriptive data showed that AR levels times decrease, while in VR levels increase (Bermejo Vidal, 2018). The results are partially in opposite with previous works that have compared VR and AR and could depend on the task to perform and on display fidelity, the accuracy and complexity of the technological systems (Irawati et al., 2008; Juan and Pérez, 2010; Khademi et al., 2013; Krichenbauer et al., 2017). Indeed, the main previous comparison studies implemented non-complex tasks in which one task was proposed at a time, such as object manipulation, and our study included activity of daily life characterized by a succession of actions and/or two tasks at a time in a rich similar real environment. For example, Khademi et al. (2013) compared an AR with VR “pick and place” task for stroke patients, showing that participants performed better in the AR condition than in VR. Möller et al. (2014) performed a study on navigation with a guidance system, showing that participants navigated in VR faster than in AR but committing more errors. Furthermore, each previous study used a different technological system with specific characteristics according to display fidelity, the accuracy, and complexity of the technological systems that could influence the results’ variability (Germine et al., 2019).

Coherent with the behavioral data are the physiological results, showing that both conditions produced individual activation with higher values in AR than VR (Bermejo Vidal, 2018). Higher physiological activation in AR could depend on the interaction system differences. More in detail, as mentioned in the description of the cooking task, in VR interaction was ensured by two hand controllers, and in AR depended on the human gaze that allowed real hand interaction with the synthetic elements.

Regarding the second aim on the sense of presence, scores between conditions showed that VR always produces a higher sense of presence than AR (Bermejo Vidal, 2018). Specifically, the higher significant results on VR SP dimension than AR could depend on the fact that VR condition is mostly unmediated. Indeed, VR created a unitary and composite synthetic environment in which the user is totally immersed without interferences from the real world and AR adds synthetic objects to the real world, being able to perceive of discordance between reality and the artificial information in the environment. Regarding the E and EV dimensions, we expected that the EV results of AR would be significantly higher than that of VR. Nevertheless, the higher score in VR could depend on the self-report measure used (ITC-SOPI) also for evaluating AR experience. Indeed, the ITC-SOPI items related to EV (5, 11, 15, 20, 27) evaluate if the environment seems natural or if was part of the real world and in AR the environment is the real world. This suggests that in the future studies a change of the scale may be needed for evaluating EV in AR. Finally, participants evaluated AR with a negative connotation with respect to RV as shown in the results and especially in SOPI-EN (NE of the ITC-SOPI) where AR has a higher score than in VR. This result seems to confirm previous results on the comparison between both conditions in situations of acrophobia, in which sense of presence was higher in VR than AR (Juan and Pérez, 2010). This result could also depend on less difficulty and greater familiarity by the subjects in using VR controllers and a feeling of greater naturalness in the interaction in AR, as mentioned in the introduction (McMahan et al., 2010).

Although the results are interesting for their possible applications in neuropsychological assessment, our study has some limitations that could affect the generalizability of the results or that may have influenced the findings. The main issues are related to the small sample size and the specific sample of healthy subjects included in this study. At the technological level, FOV and user interaction differences between the two technological systems can have generated the variability of the test scores. Futures studied are needed to investigate differences in behavioral responses comparing clinical populations and healthy subjects, as well as comparing AR and VR with other condition, such as the real condition. Furthermore, to overpass possible differences between the technological systems results, in the test design it would be important to focus more on the accuracy of the responses rather than in reaction times and also implement an individual baseline on the same or another measure using the different systems before the experimental task (Germine et al., 2019). In this way, it would be possible to consider and control system variability producing a higher generalization of the results. To conclude, VR and AR are two novels GIT with a high EV value applicable to a wide variety of research fields, so it is relevant to understand the effects of various technological systems also on neuropsychological effectiveness. Specifically, we focused on behavioral performance, physiological activation in the virtual cooking task and on the sense of presence, comparing VR and AR. We found higher results on VR than AR condition in all comparison factors.

This research represents a step toward better understanding the differences between AR and VR and opens up several new venues for future research works. In particular, we conclude that future test designs took into consideration some changes in the experimental design – adding an individual technological baseline and considering also the responses accuracy – and in the self-report scale to measure presence in AR.

## Data Availability Statement

The datasets generated for this study are available on request to the corresponding author.

## Ethics Statement

The studies involving human participants were reviewed and approved by Universitat Politècnica de València (ID: P3_02_05_18). The patients/participants provided their written informed consent to participate in this study.

## Author Contributions

IC and MA designed and directed the project. IC developed the theoretical framework and wrote the manuscript. CB carried out the experiment. IC and CB analyzed data. MA reviewed the final version of the manuscript. All authors discussed the results and contributed to the final manuscript.

## Conflict of Interest

The authors declare that the research was conducted in the absence of any commercial or financial relationships that could be construed as a potential conflict of interest.

## Footnotes

1 https://www.vive.com/eu/product/

2 https://www.microsoft.com/it-it/hololens

## References

[B1] Barratt E. S. (1959). Anxiety and impulsiveness related to psychomotor efficiency. *Percep. Motor Skills* 9 191–198. 10.2466/pms.1959.9.3.191

[B2] Bermejo Vidal C. (2018). Estudio de las funciones ejecutivas y su validez ecológica en la vida real: Realidad Aumentada (RA) vs. Realidad Virtual (RV) utilizando medidas explícitas (cuestionarios) e implícita (comportamentales y fisiológicas). Available at: http://hdl.handle.net/10251/110020

[B3] Bohil C. J. Alicea B. Biocca F. A. (2011). Virtual reality in neuroscience research and therapy. *Nat. Rev. Neurosci.* 12:752. 10.1038/nrn3122 22048061

[B4] Boucsein W. (1992). *Electrodermal activity.* New York, NY: Plenum Press, 442.

[B5] Chaytor N. Schmitter-Edgecombe M. (2003). The ecological validity of neuropsychological tests: a review of the literature on everyday cognitive skills. *Neuropsychol. Rev.* 13 181–197. 10.1023/b:nerv.0000009483.91468.fb 15000225

[B6] Chaytor N. Schmitter-Edgecombe M. Burr R. (2006). Improving the ecological validity of executive functioning assessment. *Arch. Clin. Neuropsychol.* 21 217–227. 10.1016/j.acn.2005.12.002 16554143

[B7] Chicchi Giglioli I. A. Pallavicini F. Pedroli E. Serino S. Riva G. (2015). Augmented reality: a brand new challenge for the assessment and treatment of psychological disorders. *Comput. Math. Methods Med.* 2015:862942. 10.1155/2015/862942 26339283 PMC4538767

[B8] Cipresso P. Albani G. Serino S. Pedroli E. Pallavicini F. Mauro A. (2014). Virtual multiple errands test (VMET): a virtual reality-based tool to detect early executive functions deficit in Parkinson’s disease. *Front. Behav. Neurosci.* 8:405. 10.3389/fnbeh.2014.00405 25538578 PMC4257151

[B9] Cipresso P. Chicchi Giglioli I. A. Raya M. A. Riva G. (2018). The past, present, and future of virtual and augmented reality research: a network and cluster analysis of the literature. *Front. Psychol.* 9:2086. 10.3389/fpsyg.2018.02086 30459681 PMC6232426

[B10] Culberston W. C. Zillmer E. A. (1999). *Tower of London-Drexel (TOLDX). Examiners’s Manual. Research version.* Canadá: Multi-Health Systems Inc.

[B11] Dawson M. E. Schell A. M. Filion D. L. (2007). The electrodermal system. *Handb. Psychophysiol.* 2 200–223.

[B12] De Leeuw J. R. (2015). jsPsych: a JavaScript library for creating behavioral experiments in a Web browser. *Behav. Res. Methods* 47 1–12. 10.3758/s13428-014-0458-y 24683129

[B13] Derryberry D. Reed M. A. (2002). Anxiety-related attentional biases and their regulation by attentional control. *J. Abnorm. Psychol.* 111:225. 10.1037//0021-843x.111.2.225 12003445

[B14] Díaz-Orueta U. Iriarte Y. Climent G. Banterla F. (2012). AULA: an ecological virtual reality test with distractors for evaluating attention in children and adolescents. *Vir. Real. Sci. Vis. J.* 5 1–20. 10.1177/1087054712465335 23239784

[B15] Díaz-Orueta U. Garcia-López C. Crespo-Eguílaz N. Sánchez-Carpintero R. Climent G. Narbona J. (2014). AULA virtual reality test as an attention measure: convergent validity with Conners’ continuous performance test. *Child Neuropsychol.* 20 328–342. 10.1080/09297049.2013.792332 23638628

[B16] Dunkin B. Adrales G. L. Apelgren K. Mellinger J. D. (2007). Surgical simulation: a current review. *Surg. Endosc.* 21 357–366. 10.1007/s00464-006-9072-0 17180270

[B17] Elkind J. S. Rubin E. Rosenthal S. Skoff B. Prather P. (2001). A simulated reality scenario compared with the computerized wisconsin card sorting test: an analysis of preliminary results. *CyberPsychol. Behav.* 4 489–496. 10.1089/109493101750527042 11708728

[B18] Fillmore M. T. Rush C. R. Hays L. (2006). Acute effects of cocaine in two models of inhibitory control: implications of non-linear dose effects. *Addiction* 101 1323–1332. 10.1111/j.1360-0443.2006.01522.x 16911732

[B19] Fleming T. M. Bavin L. Stasiak K. Hermansson-Webb E. Merry S. N. Cheek C. (2017). Serious games and gamification for mental health: current status and promising directions. *Front. Psychiatry* 7:215. 10.3389/fpsyt.2016.00215 28119636 PMC5222787

[B20] Folstein M. F. Folstein S. E. McHugh P. R. (1975). “Mini-mental state”: a practical method for grading the cognitive state of patients for the clinician. *J. Psychiatric Res*. 12 189–198. 10.1016/0022-3956(75)90026-6 1202204

[B21] Freeman D. Reeve S. Robinson A. Ehlers A. Clark D. Spanlang B. (2017). Virtual reality in the assessment, understanding, and treatment of mental health disorders. *Psychol. Med.* 47 2393–2400. 10.1017/s003329171700040x 28325167 PMC5964457

[B22] Garbarino M. Lai M. Bender D. Picard R. W. Tognetti S. (2014). “Empatica E3—A wearable wireless multi-sensor device for real-time computerized biofeedback and data acquisition,” in proceedings of the *2014 4th International Conference on Wireless Mobile Communication and Healthcare-Transforming Healthcare Through Innovations in Mobile and Wireless Technologies (MOBIHEALTH).* IEEE, Piscataway, NJ 39–42.

[B23] Germine L. Nakayama K. Duchaine B. C. Chabris C. F. Chatterjee G. Wilmer J. B. (2012). Is the Web as good as the lab? Comparable performance from Web and lab in cognitive/perceptual experiments. *Psychonomic Bull. Rev.* 19 847–857. 10.3758/s13423-012-0296-9 22829343

[B24] Germine L. Reinecke K. Chaytor N. S. (2019). Digital neuropsychology: challenges and opportunities at the intersection of science and software. *Clin. Neuropsychol.* 33 271–286. 10.1080/13854046.2018.1535662 30614374

[B25] Gregg L. Tarrier N. (2007). Virtual reality in mental health. *Soc. Psychiatry Psychiatr. Epidemiol.* 42 343–354. 10.1007/s00127-007-0173-4 17431528

[B26] Henry M. Joyal C. C. Nolin P. (2012). Development and initial assessment of a new paradigm for assessing cognitive and motor inhibition: the bimodal virtual-reality Stroop. *J. Neurosci. Methods* 210 125–131. 10.1016/j.jneumeth.2012.07.025 22897988

[B27] Irawati S. Hong S. Kim J. Ko H. (2008). “3D edutainment environment: learning physics through VR/AR experiences,” in *Proceedings of the 2008 International Conference on Advances in Computer Entertainment Technology.* ACM, New York, NY 21–24.

[B28] Jensen L. Konradsen F. (2018). A review of the use of virtual reality head-mounted displays in education and training. *Educ. Inform. Technol.* 23 1515–1529. 10.1007/s10639-017-9676-0

[B29] Juan M. C. Pérez D. (2010). Using augmented and virtual reality for the development of acrophobic scenarios. Comparison of the levels of presence and anxiety. *Comput. Graph.* 34 756–766. 10.1016/j.cag.2010.08.001

[B30] Khademi M. Hondori H. M. Dodakian L. Cramer S. Lopes C. V. (2013). “Comparing “pick and place” task in spatial augmented reality versus non-immersive virtual reality for rehabilitation setting,” in *Proceeding of the 2013 35th Annual International Conference of the IEEE Engineering in Medicine and Biology Society (EMBC).* IEEE, Piscataway, NJ 4613–4616 10.1109/EMBC.2013.6610575 24110762

[B31] Krichenbauer M. Yamamoto G. Taketom T. Sandor C. Kato H. (2017). Augmented reality versus virtual reality for 3d object manipulation. *IEEE Trans. Vis. Comput. Graph.* 24 1038–1048. 10.1109/TVCG.2017.2658570 28129181

[B32] Ku J. Cho W. Kim J. J. Peled A. Wiederhold B. K. Wiederhold M. D. (2003). A virtual environment for investigating schizophrenic patients’ characteristics: assessment of cognitive and navigation ability. *CyberPsychol. Behav.* 6 397–404. 10.1089/109493103322278781 14511452

[B33] Ku J. W. G. Kim J. H. Kim K. U. (2004). The development of a VR system for the cognitive & behavioral assessment of schizophrenia. *Stud. Health Technol. Inform.* 98:180. 15544267

[B34] Lessiter J. Freeman J. Keogh E. Davidoff J. (2001). A cross-media presence questionnaire: the ITC-sense of presence inventory. *Presence* 10 282–297. 10.1162/105474601300343612

[B35] Macías I. A. (2016). *Valores de Variabilidad De La Frecuencia Cardíaca En Mujeres Y Su Relación Con El Ciclo Menstrual.* Doctoral dissertation, Universidad Pablo de Olavide, Sevilla.

[B36] Martin M. M. Rubin R. B. (1995). A new measure of cognitive flexibility. *Psychol. Rep.* 76 623–626. 10.2466/pr0.1995.76.2.623

[B37] McMahan R. P. Alon A. J. D. Lazem S. Beaton R. J. Machaj D. Schaefer M. Bowman D. A. (2010). “Evaluating natural interaction techniques in video games,” in *Proceedings of the 2010 IEEE Symposium on 3D User Interfaces (3DUI)*, Piscataway, NJ: IEEE, 11–14

[B38] McMahan R. P. Bowman D. A. Zielinski D. J. Brady R. B. (2012). Evaluating display fidelity and interaction fidelity in a virtual reality game. *IEEE Trans. Vis. Comput. Graph.* 18 626–633. 10.1109/TVCG.2012.43 22402690

[B39] Miller M. A. Fillmore M. T. (2010). The effect of image complexity on attentional bias towards alcohol-related images in adult drinkers. *Addiction* 105 883–890. 10.1111/j.1360-0443.2009.02860.x 20148790 PMC2922935

[B40] Möller A. Kranz M. Diewald S. Roalter L. Huitl R. Stockinger T. (2014). “Experimental evaluation of user interfaces for visual indoor navigation,” in *Proceedings of the SIGCHI Conference on Human Factors in Computing Systems* (New York, NY: ACM), 3607–3616.

[B41] Negut A. Matu S. A. Sava F. A. David D. (2016). Virtual reality measures in neuropsychological assessment: a meta-analytic review. *Clin. Neuropsychol.* 30 165–184. 10.1080/13854046.2016.1144793 26923937

[B42] Oquendo M. A. Baca-García E. Graver R. Morales M. Montalvan V. Mann J. (2001). Spanish adaptation of the Barratt impulsiveness scale (BIS-11). *Eur. J. Psychiatry* 15 147–155. 10.1016/j.ijchp.2015.07.002 30487844 PMC6225021

[B43] Parsons T. D. Courtney C. G. Dawson M. E. (2013). Virtual reality stroop task for assessment of supervisory attentional processing. *J. Clin. Exp. Neuropsychol.* 35 812–826. 10.1080/13803395.2013.82455 23961959

[B44] Parsons T. D. (2015). Virtual reality for enhanced ecological validity and experimental control in the clinical, affective and social neurosciences. *Front. Hum. Neurosci.* 9:660. 10.3389/fnhum.2015.00660 26696869 PMC4675850

[B45] Poh M. Z. Swenson N. C. Picard R. W. (2010). A wearable sensor for unobtrusive, long-term assessment of electrodermal activity. *IEEE Trans. Biomed. Eng.* 57 1243–1252. 10.1109/TBME.2009.2038487 20172811

[B46] Pugnetti L. Mendozzi L. Attree E. A. Barbieri E. Brooks B. M. Cazzullo C. L. (1998). Probing memory and executive functions with virtual reality: past and present studies. *CyberPsychol. Behav.* 1 151–161. 10.1089/cpb.1998.1.151

[B47] Ragan E. D. Sowndararajan A. Kopper R. Bowman D. A. (2010). The effects of higher levels of presence on procedure memorization performance and implications for educational virtual environments. *Presence* 19 527–543. 10.1162/pres_a_00016

[B48] Ragan E. D. Kopper R. Schuchardt P. Bowman D. A. (2012). Studying the effects of stereo, head tracking, and field of regard on a small-scale spatial judgment task. *IEEE Trans. Vis. Comput. Graph.* 19 886–896. 10.1109/tvcg.2012.163 22868674

[B49] Rand D. Katz N. Weiss P. L. (2007). Evaluation of virtual shopping in the VMall: comparison of post-stroke participants to healthy control groups. *Disabil. Rehabil.* 29 1710–1719. 10.1080/09638280601107450 17852223

[B50] Rand D. Rukan S. B. A. Weiss P. L. Katz N. (2009). Validation of the Virtual MET as an assessment tool for executive functions. *Neuropsychol. Rehabil.* 19 583–602. 10.1080/09602010802469074 19058093

[B51] Reimers S. Stewart N. (2015). Presentation and response timing accuracy in Adobe Flash and HTML5/JavaScript web experiments. *Behav. Res. Methods* 47 309–327. 10.3758/s13428-014-0471-1 24903687 PMC4427652

[B52] Rizzo A. A. Buckwalter J. G. Bowerly T. Van Der Zaag C. Humphrey L. Neumann U. (2000). The virtual classroom: a virtual reality environment for the assessment and rehabilitation of attention deficits. *CyberPsychol. Behav.* 3 483–499. 10.1089/10949310050078940 16400254

[B53] Rizzo A. A. Schultheis M. Kerns K. A. Mateer C. (2004). Analysis of assets for virtual reality applications in neuropsychology. *Neuropsychol. Rehabil.* 14 207–239. 10.1080/09602010343000183

[B54] Rizzo A. A. Bowerly T. Buckwalter J. G. Klimchuk D. Mitura R. Parsons T. D. (2009). A virtual reality scenario for all seasons: the virtual classroom. *CNS Spectr.* 11 35–44. 10.1017/s1092852900024196 16400254

[B55] Saposnik G. Mamdani M. Bayley M. Thorpe K. E. Hall J. Cohen L. G. (2010). Effectiveness of virtual reality exercises in STroke rehabilitation (EVREST): rationale, design, and protocol of a pilot randomized clinical trial assessing the Wii gaming system. *Int. J. Stroke* 5 47–51. 10.1111/j.1747-4949.2009.00404.x 20088994 PMC4880012

[B56] Seymour N. E. (2008). VR to OR: a review of the evidence that virtual reality simulation improves operating room performance. *World J. Surg.* 32 182–188. 10.1007/s00268-007-9307-9 18060453

[B57] Sequeira H. Hot P. Silvert L. Delplanque S. (2009). Electrical autonomic correlates of emotion. *Int. J. Psychophysiol.* 71 50–56. 10.1016/j.ijpsycho.2008.07.009 18723054

[B58] Slater M. (2009). Place illusion and plausibility can lead to realistic behaviour in immersive virtual environments. *Philos. Trans. R. Soc. Lond.* 364 3549–3557. 10.1098/rstb.2009.0138 19884149 PMC2781884

[B59] Slater M. Steed A. (2000). A virtual presence counter. *Presence* 9 413–434. 10.1162/105474600566925

[B60] Suso-Ribera C. Fernández-Álvarez J. García-Palacios A. Hoffman H. G. Bretón-López J. Banos R. M. Botella C. (2018). Virtual reality, augmented reality, and in vivo exposure therapy: a preliminary comparison of treatment efficacy in small animal phobia. *Cyberpsychol. Behav. Soc. Netw.* 22 31–38. 10.1089/cyber.2017.0672 30335525 PMC6352498

[B61] Stroop J. R. (1992). Studies of interference in serial verbal reactions. *J. Exp. Psychol.* 121:15 10.1037//0096-3445.121.1.15

[B62] Tang A. Biocca F. Lim L. (2004). “Comparing differences in presence during social interaction in augmented reality versus virtual reality environments: an exploratory study,” in *Proceedings of PRESENCE 2004, 7th Annual International Workshop on Presence*, eds Raya M.A. Solaz B.R Technical University of Valencia, Valencia 204–208.

[B63] Valmaggia L. R. Latif L. Kempton M. J. Rus-Calafell M. (2016). Virtual reality in the psychological treatment for mental health problems: an systematic review of recent evidence. *Psychiatry Res.*, 236 189–195. 10.1016/j.psychres.2016.01.015 26795129

[B64] Ventura S. Baños R. M. Botella C. (2018). *Virtual and Augmented Reality: New Frontiers for Clinical Psychology, State of the Art Virtual Reality and Augmented Reality Knowhow.* London: IntechOpen.

